# Mutation analysis and characterization of ATR sequence variants in breast cancer cases from high-risk French Canadian breast/ovarian cancer families

**DOI:** 10.1186/1471-2407-6-230

**Published:** 2006-09-29

**Authors:** Francine Durocher, Yvan Labrie, Penny Soucy, Olga Sinilnikova, Damian Labuda, Paul Bessette, Jocelyne Chiquette, Rachel Laframboise, Jean Lépine, Bernard Lespérance, Geneviève Ouellette, Roxane Pichette, Marie Plante, Sean V Tavtigian, Jacques Simard

**Affiliations:** 1 Cancer Genomics Laboratory, Oncology and Molecular Endocrinology Research Centre, Centre Hospitalier Universitaire de Québec and Laval University, Québec, G1V 4G2, Canada; 2 Unité Mixte de Génétique Constitutionnelle des Cancers Fréquents, Hospices Civils de Lyon/Centre Léon Bérard, Lyon, France; 3 Centre de cancérologie Charles Bruneau, Ste-Justine Hospital, Montréal, Canada; 4 Service de gynécologie, Centre Hospitalier Universitaire de Sherbrooke, Fleurimont, Canada; 5 Clinique des maladies du sein Deschênes-Fabia, Hôpital du Saint-Sacrement, Québec, G1S 4L8, Canada; 6 Service de médecine génétique, CHUQ, Pavillon CHUL, Québec, G1V 4G2, Canada; 7 Centre hospitalier régional de Rimouski, Rimouski, G5L 5T1, Canada; 8 Service d'hémato-oncologie, Hôpital du Sacré-Cœur, Montréal, Canada; 9 Service de gynécologie, CHUQ, L'Hôtel-Dieu de Québec, Québec, G1R 2J6, Canada; 10 Unit of Genetic Cancer Susceptibility, International Agency for Research on Cancer, World Health Organization, Lyon, France; 11 Canada Research Chair in Oncogenetics, Department of Anatomy and Physiology, Laval University, Québec, Canada

## Abstract

**Background:**

Ataxia telangiectasia-mutated and Rad3-related (ATR) is a member of the PIK-related family which plays, along with ATM, a central role in cell-cycle regulation. ATR has been shown to phosphorylate several tumor suppressors like BRCA1, CHEK1 and TP53. *ATR *appears as a good candidate breast cancer susceptibility gene and the current study was designed to screen for *ATR *germline mutations potentially involved in breast cancer predisposition.

**Methods:**

*ATR *direct sequencing was performed using a fluorescent method while widely available programs were used for linkage disequilibrium (LD), haplotype analyses, and tagging SNP (tSNP) identification. Expression analyses were carried out using real-time PCR.

**Results:**

The complete sequence of all exons and flanking intronic sequences were analyzed in DNA samples from 54 individuals affected with breast cancer from non-BRCA1/2 high-risk French Canadian breast/ovarian families. Although no germline mutation has been identified in the coding region, we identified 41 sequence variants, including 16 coding variants, 3 of which are not reported in public databases. SNP haplotypes were established and tSNPs were identified in 73 healthy unrelated French Canadians, providing a valuable tool for further association studies involving the ATR gene, using large cohorts. Our analyses led to the identification of two novel alternative splice transcripts. In contrast to the transcript generated by an alternative splicing site in the intron 41, the one resulting from a deletion of 121 nucleotides in exon 33 is widely expressed, at significant but relatively low levels, in both normal and tumoral cells including normal breast and ovarian tissue.

**Conclusion:**

Although no deleterious mutations were identified in the *ATR *gene, the current study provides an haplotype analysis of the *ATR *gene polymorphisms, which allowed the identification of a set of SNPs that could be used as tSNPs for large-scale association studies. In addition, our study led to the characterization of a novel Δ33 splice form, which could generate a putative truncated protein lacking several functional domains. Additional studies in large cohorts and other populations will be needed to further evaluate if common and/or rare *ATR *sequence variants can be associated with a modest or intermediate breast cancer risk.

## Background

All common cancers show some degree of familial clustering [1]. Most of the familial aggregation, especially in breast cancer [2], results predominantly from inherited susceptibility [3]. Linkage studies in the 1990s led to the discovery of several predisposition genes associated with many rare familial cancer syndromes, thus providing fundamental insights into various pathways of carcinogenesis [4]. Nevertheless, this approach has mainly been limited to genes with relatively rare, highly penetrant alleles, for several reasons, such as a lack of power to detect alleles conferring modest or moderate risks that are believed to be involved in common cancers [1,5-7]. Analyses of risk attributable to such alleles in the known breast cancer susceptibility genes (e.g. *BRCA1, BRCA2, TP53, PTEN, ATM*) suggest they are responsible for ~25% of the familial component of breast cancer risk [6,8,9]. The number and properties of genetic variants that account for the remaining 75% of inherited risk are largely unknown. It has been proposed that a complex polygenic model is the best explanation for this missing genetic risk [10,11] and perhaps the majority of breast cancers arise in a susceptible minority of women [2,12].

Under the Common Variant/Common Disease (CV/CD) model, disease susceptibility is suggested to result from the joint action of several common variants, with unrelated affected individuals sharing a substantial proportion of disease alleles [13-15]. The alternative is the heterogeneity hypothesis, which maintains that genetic susceptibility to common disease is caused by many different rare genetic variants, with a relatively large effect produced by each allele [16-19]. If most cancer susceptibility is related to fundamental processes of cellular control, rare alleles might turn out to be the more important component and should be detectable by linkage analysis and/or the candidate gene re-sequencing approach [5,6].

The central role of *BRCA1 *and *BRCA2 *genes in DNA repair, recombination, cell cycle control and transcription [20,21] has led to the investigation of the implication of several similarly acting genes in breast and/or ovarian cancer predisposition, including *ATM *(Ataxia telangiectasia-mutated) [22-27], *CHEK2 *[28,29], *TP53 *[30], *PTEN *[31], *STK11 *[32] and a few other genes involved in DNA repair [33]. Ataxia-telangiectasia-mutated and Rad3-related (ATR) is a member of the phosphatidyl inositol-kinase (PIK)-related family which plays, along with ATM, a central role in cell-cycle regulation, by transducing DNA damage signals to downstream effectors of cell-cycle progression [34]. In response to double-strand breakage, stalled replication forks or DNA adducts, ATR complexed with ATR-interacting protein (ATRIP) is recruited and then phosphorylates a number of proteins involved in DNA damage, including H2AX, 53BP, TP53, NBS1 and CHEK1 [35-38], thereby activating cell checkpoints, DNA repair or apoptosis. ATR is also able to bind to Rad17 and BRCA1 and to associate with components of the nucleosome remodeling and deacetylating complex [39-41]. Furthermore, ATR has recently been shown to interact with the Fanconi Anemia complex [42], which growing number of evidences link to the two *BRCA *genes [[21], for review see [43]]. A recent study has also demonstrated that the Mre11/Rad50/NBS1 (MRN) complex, a central component in the cellular response to ionizing radiations and other causes of double-strand breaks, is required for ATR-dependant phosphorylation mechanisms of the protein Smc1 (Structural maintenance of chromosomes 1) [44]. ATR knockout studies showed that ATR is essential for somatic cell growth and genomic integrity in the embryo and that its deletion leads to genomic disruption and early embryonic lethality in mice [45,46]. Moreover, it has been reported that disruption of the *ATR *gene leads to an increase in the incidence of large benign tumors in heterozygotes, possibly indicating that deficiency in ATR affects the rate of tumor initiation [45].

Based on the major role of ATR in cellular response to DNA damage and its multiple interactions with several proteins such as BRCA1 [40,47], *ATR *represents an attractive candidate gene to potentially explain a fraction of the remaining breast cancer susceptibility. The current study was designed to assess the possible involvement of *ATR *germline mutations in breast cancer susceptibility. For this purpose, the complete sequence of the 47 exons and flanking intronic sequences of the *ATR *gene were analyzed in DNA samples from individuals affected with breast cancer from non-related *BRCA1- *and *BRCA2*-negative high-risk French Canadian breast/ovarian families.

## Methods

### Ascertainment of families and DNA extraction

The recruitment of high-risk French Canadian breast and/or ovarian families started in 1996 through a research project, which thereafter evolved in a large ongoing interdisciplinary research program designated INHERIT BRCAs. More details regarding ascertainment criteria, experimental and clinical procedures as well as the INHERIT BRCAs research program have been described elsewhere [48-52]. A major component was to identify and characterize *BRCA1 *and *BRCA2 *mutations in French Canadian high-risk families (CGL cohort) [52].

Subsequently, another component was designed for the "Localization and identification of new breast cancer susceptibility loci/genes". Ethics approval for this latter study was also obtained from the different institutions participating in this research project and each participant knowing their inconclusive *BRCA1/2 *test results status had to sign a specific informed consent for their participation in this component. A subset of 54 high-risk French Canadian breast/ovarian cancer families were recruited in the present study according to the following ascertainment criteria 1) three or more breast cancer cases diagnosed before the age of 65 (48 families), 2) two or more breast cancer cases (<65) if one breast cancer was diagnosed before 45 years (5 families), 3) or when there was a strong family history of breast/ovarian cancer (e.g. daughter-mother-grand-mother) (1 family). All participants had to be at least 18 years of age and mentally capable. The diagnoses of breast and/or ovarian cancer were confirmed by obtaining a pathology report, and when two or more subjects were available within a family, the youngest subject was systematically chosen for this study. The mean age at diagnosis of these 54 subjects affected with breast cancer was 45.5 years old (30–59 years), while 46 of them have been diagnosed before 50 year of age and 11 were affected by more than one breast cancer case. The analysis of the breast cancer history revealed that 15 (28%), 18 (33%) and 19 (35%) families included 1–2, 3 or ≥ 4 case(s) in at most 2^nd ^degree relatives, respectively. When including all breast cancer cases in the family history occurring in at most 3^rd ^degree relatives from the index case, 10 (18%), 16 (30%) and 28 (52%) families have 1–2, 3 or ≥ 4 case(s), respectively.

The *BRCA1/2 *status of each participant was previously assessed [52]. Briefly, to this day, genomic DNA samples have been first tested for a panel of 29 mutations, including 26 truncating mutations and 3 unclassified variants (two missense mutations and one in-frame deletion), observed and/or reported in the French Canadian population [52]. Thereafter, DNA samples of individuals included in this study were sent to Myriad Genetic Laboratories (Salt Lake City, Utah, USA) for full-length *BRCA1/2 *sequencing following their *Comprehensive BRACAnalysis ^®^-BRCA1 and BRCA2 gene sequence analysis for susceptibility to breast and ovarian cancer test*, with the exception of 9 subjects for which DNA samples from another affected individual of the family (n = 7) or unaffected parents of cancer cases (n = 2) were sent to Myriad as previously described [49,50,52]. Evidence of the absence of genomic rearrangements in *BRCA1/2 *genes was thereafter investigated by Multiplex Ligation-dependant Probe Amplification (MLPA) for 45 of the 54 subjects and *BRCA1/2 *Southern analysis for 32 of the 54 individuals. For seven of the remaining subjects, MLPA was performed on another individual of the family [53], while for two subjects this analysis was not performed.

Genomic DNA from 73 healthy unrelated French Canadian women was obtained from Dr Damian Labuda at the Centre de cancérologie Charles Bruneau, Hôpital Ste-Justine, Montreal, Canada. The individuals who provided these samples were recruited on a non-nominative basis, in the framework of long-term studies aiming the characterization of the genetic variability in human populations, approved by the Institutional Ethic Review Board. DNA from peripheral blood was isolated by conventional methods, either phenol-chlorophorm or using Gentra kits (Minneapolis, MN, USA). The mean age of these individuals was 45.2 years old; 2 (2.7%), 26 (35.6%), 23 (31.5), 17 (23.3%) and 5 (6.8%) of them were between 25–29, 30–39, 40–49, 50–59 and 60–69 year of age, respectively.

The validation group comprised 46 BRCA1/2-negative breast cancer proband cases of French origin belonging to multiple-case breast cancer families from the following sources: high-risk breast cancer only and breast/ovarian cancer families referred for genetic testing at the Department of Preventive Medicine at Creighton University School of Medicine, Omaha, NE, and at the cancer genetic counseling unit at Centre Léon Bérard, Lyon, France, and a population-based study including women diagnosed with breast cancer below age 46 years, recruited through the Rhône region cancer registry, France. The cancer status of index cases was confirmed through pathology reports. Cancers reported in relatives were verified through pathology reports, hospital records and death certificates. Index cases have been screened for mutations in *BRCA1 *and *BRCA2 *[54,55]. All subjects provided written informed consent for participation in the study. Approval for the study was obtained from the International Agency for Research on Cancer (IARC) ethics committee.

The mean age at diagnosis of these 46 French subjects affected with breast cancer was 39.7 years old (19–61 years); 44 of them have been diagnosed before 50 year of age and five were affected by more than one cancer case. The analysis of the breast cancer history revealed that 23 (50%), 9 (20%) and 14 (30%) families included 1–2, 3 or ≥ 4 case(s) in at most 2^nd ^degree relatives, respectively. When including all breast cancer cases in the family history occurring in at most 3^rd ^degree relatives from the index case, 12 (26%), 14 (31%) and 20 (43%) families have 1–2, 3 or ≥ 4 case(s), respectively.

### PCR amplification, mutation analysis and variant characterization

The intron-exon boundaries of the *ATR *gene were determined by aligning GenBank mRNA records (NM_001184) with genomic sequence records (NC_000003). *ATR *spans approximately 130 kb and is composed of 47 exons (3q22-q24: 143650778-143780349). PCR amplicons using primers designed by the Primer Express 2.0 software (Applied Biosystems, Foster City, CA, USA) covered the entire mRNA encoding portions and flanking intronic sequences from genomic DNA. Forty primer pairs were used to amplify fragments ranging in size from 351 bp to 1385 bp, which were sequenced with primers also indicated in the table [see Additional file 1]. *ATR *direct sequencing was performed on an ABI3731 automated sequencer using version 3.1 of the Big Dye fluorescent method according to the manufacturer's instructions (Applied Biosystems, Foster City, USA). Sequence data were analyzed using the Staden preGap4 and Gap4 programs.

### LD analysis, haplotype estimation and tagging SNP selection (tSNP)

To estimate the pattern of linkage disequilibrium (LD), all 41 SNPs identified in our breast cancer case series have been genotyped. The LDA program [56] was used to calculate pairwise LD for each SNP pair. Lewontin's |D'| was used as a measure of LD between SNPs [57,58].

Haplotype analysis was performed using PHASE 2.1.1 software [59,60]. This program (PHASE) estimates haplotype frequencies with a Bayesian-based algorithm and then uses a permutation test to determine the significance of differences in inferred haplotypes between cases and controls. All association tests were run under default conditions, with 1000 permutations. Haplotype frequencies were estimated using the SNPs with minor allele frequency (MAF) ≥ 5% identified in both sample series (cases and controls). Haplotype blocks were identified using genotyping data from control individuals as well as using HapMap data from the CEPH cohort [61] using the Haploview [62,63] software. Tagging SNPs (tSNPs) from each LD block were then identified using the same software. Splice site prediction scores were evaluated using SSPNN [64] while protein alignment was performed using ClustalW [65].

### RNA isolation from cell lines and normal tissue samples

Total RNA was extracted using TRI Reagent^® ^(Molecular Research Center inc, Cincinnati, OH, USA) according to the manufacturer's instructions as previously described [66] from 1) EBV-transformed B-lymphoblastoid cell lines from the 54 cases used for our mutation screening; 2) nine cancer cell lines obtained from the American Type Culture Collection (ATCC) including, two estrogen receptor (ER)-negative breast cancer cell lines (BT-20 and MDA-MB-231), four ER+ breast cancer cell lines (BT-474, CAMA-1, MCF7 and ZR75) and three prostate cancer cell lines (22RV1, LNCaP, PC3); and 3) the HaCat human skin keratinocyte cell line which was generously supplied by Dr. N.E. Fusenig (German Cancer Research Center, Heidelberg, Germany) [67,68]. Total RNA samples from normal tissues were purchased directly either from Stratagene (breast and ovary) (La Jolla, CA, USA), BioChain Institute Inc. (leukocyte) (Hayward, CA, USA), or Clontech (all other normal tissue samples) (Palo Alto, CA, USA). RNA samples were then processed as previously described [66]. Thereafter, reverse transcription of 2.5 μg of standardized RNA samples was performed using 250 ng random hexamers and 200 U of SuperScript™ II RNase H^- ^Reverse Transcriptase (Invitrogen, Carlsbad, CA, USA) following the supplier's protocol.

### Characterization of the Δ33 and insΔInt41 alternative splice transcripts

#### Δ33 alternative splice transcript

In order to investigate if the SNP c.5739-4del9+T may lead to alternative splice transcript(s), a PCR reaction was performed, using the forward primer (5'- GCAGATGGAAAATCTACAACATGGA) and reverse primer (5'- TGATTTCCATATTGTAGAGATCTGCCA) designed to allow amplification of a specific *ATR *cDNA fragment spanning nucleotides 5479 to 6348 of the wild-type mRNA, with cDNA samples from immortalized cell lines from two homozygous and one heterozygous individuals for this variant, as well as two wild-type individuals. PCR product lengths were analyzed by migration on 1.5% agarose gel and sequenced in both orientations. Thereafter amplified *ATR *cDNA fragments were subcloned in the pCRII vector (TA cloning kit from Invitrogen) according to the manufacturer's instructions. After growing colonies and extracting the plasmid DNA samples using the GFX Micro Plasmid Prep Kit (Amersham), sequencing of each amplicon was performed as described above using the forward amplification primer. The only alternative splice transcript observed, designated Δ33, yielded to a 749 bp PCR product, while the wild-type fragment length was 870 bp.

Then, to further investigate for the presence of alternative splice transcript(s), which could be associated with the SNP c.5739-4del9+T, a series of primers were designed on exon-exon junctions to amplify different cDNA fragments covering exons 30–38 using the same cDNA samples described above. Four forward primers were designed on exon junctions 29–30, 30–31, 31–32 and 32–33 (F29-30: 5'-GAACCAGACCAGATCATTCATTA-3', F30-31: 5'-TAACAGGTCCGAGTGGACAGA-3', F31-32: 5'-CAGCAGATGGAAAATCTACAACAT-3', F32-33: 5'-GTGAGATTGCACATGTTATGTGAG-3') and five reverse primers were designed on exon junctions 33–34, 34–35, 35–36, 36–37 and 37–38 (R33-34: 5'-TTGTAATCTGGTCTTTTGTTGAGG-3', R34-35: 5'-TGGTGAACATCACCCTTGGAC-3', R35-36: 5'-CACGCGGTCACATCCTTATATT-3', R36-37: 5'-CCATATTGTAGAGATCTGCCAAAAT-3', R37-38: 5'-CCAGCTTTTTCCCATTCATAT-3'). Each forward primer was used in combination with each reverse primer, thus resulting in twenty distinct overlapping PCR fragments.

Given that the intronic c.5739-4del9+T variant is located at the splice acceptor site of exon 34, four additional reverse and forward primers were designed on the putative Δ33-35 and 33–35 exon junctions to verify if there is a splice variant resulting specifically from exon 34 skipping (FΔ33-35: 5'-CCAGCATTCTCCAGGGTGA-3', F33-35: 5'-TACTAAGCCTCAACAAAAGGGTGA-3', RΔ33-35: 5'-CTGGTGAACATCACCCTGGA-3', R33-35: 5'-GCCTGGTGAACATCACCCTTT-3'). PCR reactions were performed for 40 cycles using cDNA samples from c.5739-4del9+T variant carriers, as well as two wild-type individuals, and analyzed as described above.

#### insΔInt41 alternative splice transcript

In order to investigate whether the SNP c.7041+8G/A may lead to alternative splice transcript(s), a strategy similar to that described above was used. Two forward primers were designed on exon junctions 38–39 and 39–40 (F38-39: 5'-GTCAAAGTCATCTTATCCCATGC-3', F39-40: 5'-AACCGGTTGATGGAAGTAGTTCCA-3') and two reverse primers were designed on exon junctions 43–44 and 44–45 (R43-44: 5'-CTACTACTGTACCATGATGTAGGATCAG-3', R44-45: 5'-GTTTCTCCCTTATTGAAAAGACAATTG-3'. Furthermore, two additional reverse and forward primers were designed specifically on putative exon41-intron41 (Forward) and intron41-exon42 (Reverse) junctions to detect a splice variant in which the exon 41–42 junction could have been altered (F41-int41: 5'-CAATTCCTTGATTAATAAGGTTG-3', R42-int41: 5'-CTCTGCATCTTTTCTTAAGCACTCA-3'). The F41-int41 primer was used in combination with R43-44 and R44-45 while the R42-int41 primer was used in combination with F38-39 and F39-40. PCR reactions were performed for 40 cycles using cDNA samples from the c.7041+8G/A heterozygote carrier, as well as two wild-type individuals and PCR products have been analyzed as described in the previous sub-section. The structure of the alternative splice variant (r.[7041_7042ins7041+1_7041+441], designated in the current study as insΔInt41, amplified using either F41-int41 or R42-int41 with appropriate external primers was confirmed by sequencing subcloned PCR products.

### Quantitative RT-PCR (QRT-PCR) for the Δ33 splice transcript

#### Primer and Taqman probe design

Primers and Taqman probes for *ATR *wild-type and Δ33 splice transcript fragments were designed with the assistance of the Primer Express 2.0 software. The primer and probe sequences were; *ATR *wild-type: forward primer: 5'-AACTGGGTAGCTCGACTAGAAATGA-3', reverse primer: 5'-TTCATTGTAATCTGGTCTTTTGTTGAG-3', probe: 5'-FAM-TCCGGAGAGCCAGGA-3'; *ATR *splice transcript: forward primer: 5'-GAGGCTCCTACCAACGAGGAT-3', reverse primer: 5'-TCATTGTAATCTGGTCTGGAGAATG-3', probe: 5'-FAM-TGGTTTGATGCTATGCTC-3'. All probes were purchased from Applied Biosystems, as were primers and probe for 18S RNA, which was used as endogenous control gene.

#### Subcloning and standard curves

cDNA samples prepared from RNA extracted from immortalized cell lines were used to amplify by PCR three fragments corresponding to the *ATR *cDNA region spanning nucleotides 5748–5858 (NM_001184), the *ATR *splice transcript spanning nucleotides 5626–5857 and including a deletion of the last 121 nucleotides of exon 33, and a fragment spanning nucleotides 450–619 of 18S RNA. The fragments were thereafter subcloned in the pCR^®^II vector (TA Cloning^® ^from Invitrogen, Carlsbad, CA, USA) according to the manufacturer's instructions. To ensure amplification specificity, the reverse primer for the wild-type *ATR *fragment was designed on the junction between exons 33–34 while the reverse primer specific to the alternate splicing transcript was placed on the new exon 33–34 junction created by the exon 33 3'-deletion. Plasmid constructions were amplified and purified using Plasmid Maxi Kit (Qiagen, Mississauga, ON, Canada). Specific standard curves were generated by making 2-fold serial dilutions of plasmid constructions in the appropriate range for each quantitation assay.

#### QRT-PCR assays

QRT-PCR assays were performed in triplicate on an ABI 7900 Sequence Detection System (Applied Biosystems) as previously described [66]. For all the assays, a reaction mixture was prepared in a final volume of 10 μl with 1X Taqman Master Mix Buffer (Applied Biosystems) which included Taq Gold polymerase, 200 nM of Taqman^® ^probe, 900 nM of each primer for *ATR *wild-type and alternative transcript assays or 50 nM of each primer for 18S RNA assays, and cDNA samples reverse-transcribed from total RNA. The amount of cDNA used for quantitation was 15 ng for wild-type, 150 ng for splice transcript and 1.5 ng for 18S RNA.

### QRT-PCR for the insΔInt41 splice transcript

cDNA corresponding to 20, 200, 500 ng and 1 μg of total RNA coming from the immortalized cell lines of the c.7041+8G/A heterozygote carrier, as well as nine wild-type individuals, were used to perform fluorescent-based real-time PCR quantification using the Light Cycler Real-Time PCR apparatus (Roche Inc, Nutley, NJ). The primer sequences were; *ATR *wild-type: forward primer: 5'- GTCATATACACTCCCTTTTCTTTA-3', reverse primer: 5'-GTCATATACACTCCCTTTTCTTTA-3'; *ATR *splice transcript: forward primer: 5'- ACCATTTACTTTGTCTCCATTA-3', reverse primer: 5'-GTCATATACACTCCCTTTTCTTTA-3'. Expression analyses were then carried out as previously described [69].

## Results

### ATR mutation analysis and variant characterization

Although no truncating mutation was found in the *ATR *coding region of our French Canadian breast cancer cases, we identified 41 variants in *ATR *exonic and flanking intronic sequences (Table 1). These included 16 nucleotide substitutions in the exons, 6 of which resulted in amino acid changes, and 25 variants in the intronic regions, consisting of 23 nucleotide substitutions, one deletion and one insertion-deletion. Of the 41 variants, 21 were novel while 20 were reported in the single nucleotide polymorphism (dbSNP) database. Eight of the identified variants were very common polymorphisms with MAF around 40%. Of these, three were intronic variants, one was located in the 3'-UTR region and four were coding polymorphisms, only one of which caused an amino acid change. This latter variant, Thr211Met, is located in exon 4 and was previously reported in the dbSNP database. Twenty-five of the identified variants had MAF around or below 5% (Table 1). Of these, 14 polymorphisms were found exclusively in 3 individuals (s1-3-6-7-13-16-17-18-19-23-24-31-32-33), and 9 were observed only once. The six coding variants causing an amino acid change were genotyped in an independent European Caucasian validation group consisting of 46 unrelated breast cancer cases originating from high-risk non-BRCA1/2 families (Table 2). Frequencies were similar in both cohorts as well as with frequencies reported in the dbSNP database. Comparison of most common variants with those described in the recent study in Finnish families shows no notable differences in carrier frequency, with the exception of the c.268C>T variant which has a carrier frequency of 12.7% in the Finnish families and which was not observed in our cases [70]. This may be attributed to population-specific differences.

Genotype and MAF were determined in cases and controls, both of French Canadian origin, for all coding variants as well as the intronic variants showing a MAF ≥ 5%. As indicated in Table 3, most genotype distributions significantly deviating from those expected under Hardy-Weinberg Equilibrium (HWE) involved rare SNPs (i.e. s6-7-13-16-18-32-33) found exclusively in 3 cases who seem to carry a specific allele.

### Conservation of human ATR residues

**Figure 1:**
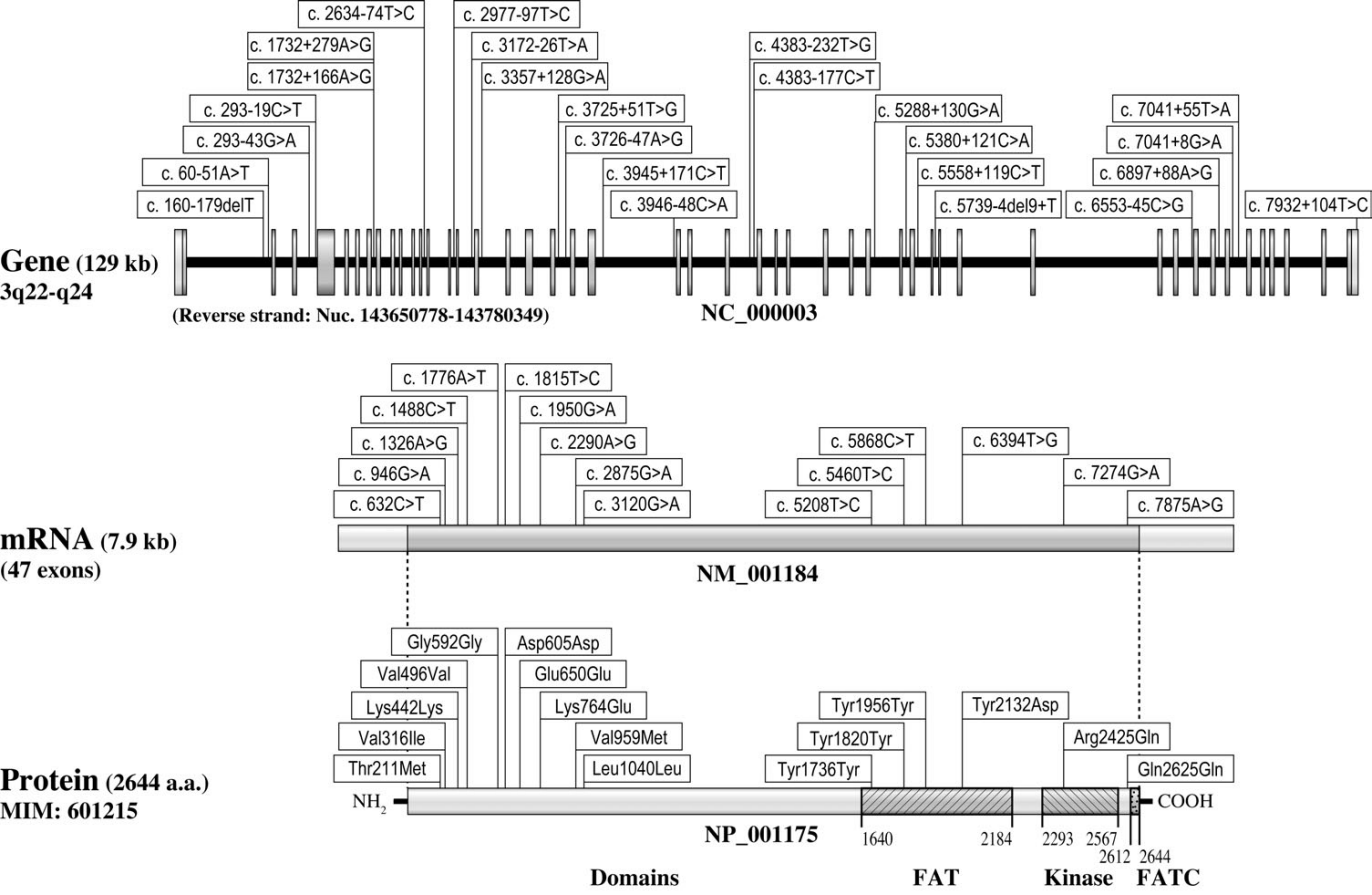
Genomic structure of the human ATR gene. The sizes of exons and introns are represented by proportionaly sized dark boxes and solid lines, respectively. The 5' and 3' untranslated regions are indicated by light boxes in the gene and mRNA structure. The GenBank accession number corresponding to the ATR gene contig, the mRNA and the protein are indicated below the nucleotide and protein sequence. The domains involved in the ATR protein activity are illustrated in the protein structure. All polymorphisms are indicated in open boxes while amino acid nomenclature is also represented.

Among exonic variants resulting in amino acid substitutions, c.6394T>G (Tyr2132Asp) was located in the FAT (FRAP/ATM/TRRAP) domain and c.7274G>A (Arg2425Gln) was located in the PI3Kc (phosphoinositide 3-kinase related catalytic) domain (Figure 1) while the remaining amino acid substitutions were located outside catalytic domains. Comparison of missense substitutions was performed across relevant species in order to obtain a more representative prediction of the importance of specific residues on protein function. Alignment of *ATR *orthologue sequences illustrated in Table 4, revealed that, with the exception of Thr211Met and Val959Met, which are non-conserved residues (Thr211Met being conserved only in *Pan troglodytes *and *Canis familiaris*), Val316Ile and Lys764Glu are conserved in *Pan troglodytes *and in more distant species such as *Canis familiaris*, *Mus musculus*, *Xenopus laevis *and *Fugu rubribes*. Since Val316 and Lys764 residues are invariant from human to fish, this could suggest that these positions are under strong functional constraint. Tyr2132 and Arg2425 are only conserved in higher species, namely *Pan troglodytes *and *Canis familiaris*, respectively.

### LD analysis

**Figure 2:**
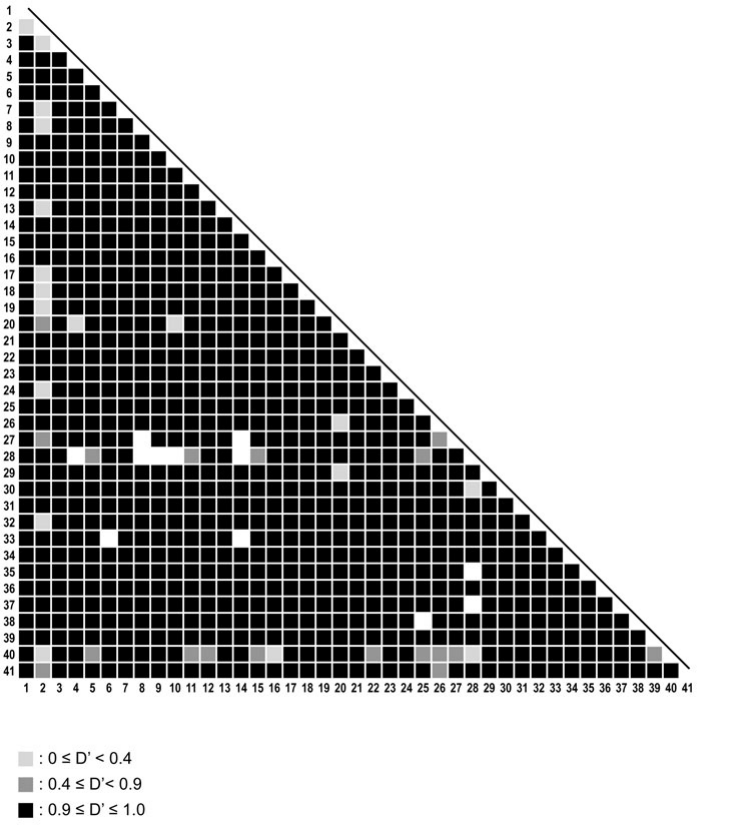
Pairwise linkage disequilibrium (LD) measures of |D'| for the 41 SNPs identified in our breast cancer cases series. All SNPs are denoted numerically with reference to Table 1.

A graphical representation of the pairwise LD between all 41 SNPs identified in cases, as measured by Lewontin's |D'|, is shown in Figure 2. As demonstrated, the majority of SNPs are in strong LD with each other. Complete LD was found between the two most distantly separated intragenic SNPs (SNPs 1 and 41, inter-marker distance ~130 kb, |D'| = 1) which suggested that LD at the ATR locus did not decrease significantly with distance. For a few associations involving SNP2, the weaker LD values range from 0.113 to 0.441. Indeed, the only pair of adjacent SNPs displaying weak LD is SNP2 in association with SNP1 and SNP3 (D' = 0.118 and 0.113, respectively) and SNP40 with SNP39 (D' = 0.467). The SNP40 also displayed a large spectrum of LD values, interestingly all of these SNPs involved with SNP40, show a MAF >5%.

### Haplotype analysis and tSNP identification

To reduce genotyping costs and efforts in future association studies, it is useful to identify tSNPs that would represent the majority of observed haplotypes, but with minimal reduction in power to detect a possible association. When estimating the *ATR *haplotypes reconstructed from the 17 SNPs having a MAF ≥ 5% in breast cancer cases, 24 haplotypes were identified from our data. Of these, 13 haplotypes exhibited a combined frequency ≥1%, which represent 95% of all haplotypes estimated by PHASE in cases and controls combined (data not shown).

**Figure 3:**
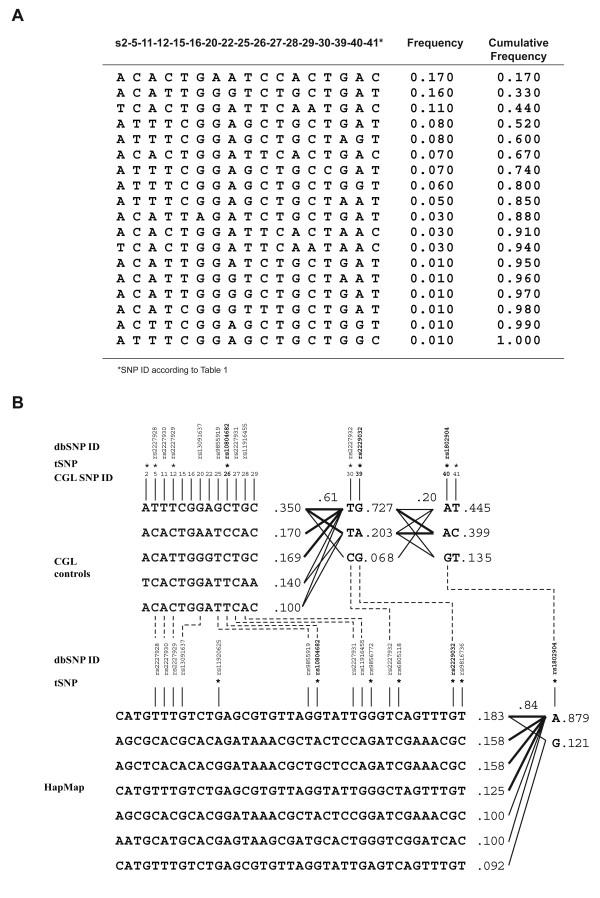
Panel A: The Table denotes the frequencies, using PHASE, of haplotypes using SNPs having a MAF higher than 5% estimated in controls. Panel B: Haplotype blocks predicted using SNPs identified in control series showing a MAF higher than 5% (17 SNPs). tSNPs identified on a block-by-block basis are denoted with an asterisk (*) above the SNP number. Population haplotype frequencies are displayed on the right of each haplotype combination while the level of recombination is displayed above the connections between two blocks. Thick connections represent haplotypes with frequencies higher than 10% while frequencies below 10% are represented by thin lines.

Thereafter, in order to identify *ATR *tSNPs useful for well-powered studies using larger sample sets, genotypes of 17 coding and intronic SNPs showing a MAF ≥5% in healthy individuals have been used for further haplotype analyses (Figure 3, Panel A). Genotyping data from controls only were purposely selected as they could well be more representative of the French Canadian population. When only haplotypes displaying a MAF ≥5% are used, 85% of all estimated haplotypes are represented by the tSNPs identified.

The identification of tSNPs was then carried out in two subsequent steps, firstly by determining haplotype blocks, followed by identification of tSNP in each LD block. Based on the algorithm from Gabriel *et al*. [71], three LD blocks have been identified in the French Canadians by the Haploview software (expectation maximisation algorithm) (Figure 3, Panel B). Thereafter, considering haplotypes having a MAF ≥5%, 8 tSNPs have been identified in the 3 LD blocks, namely SNPs 2, 5, 12 and 26 found in block 1, SNPs 30 and 39 in block 2, while block 3 consists of SNPs 40 and 41. Furthermore, seven tSNPs clustered in two LD blocks were selected in the *ATR *gene using the HapMap data from the CEPH/CEU cohort. It is of interest to note that three tSNPs have been tagged in both the French Canadian and the CEPH/CEU sample sets.

### Splicing consensus sequence analysis

**Figure 4:**
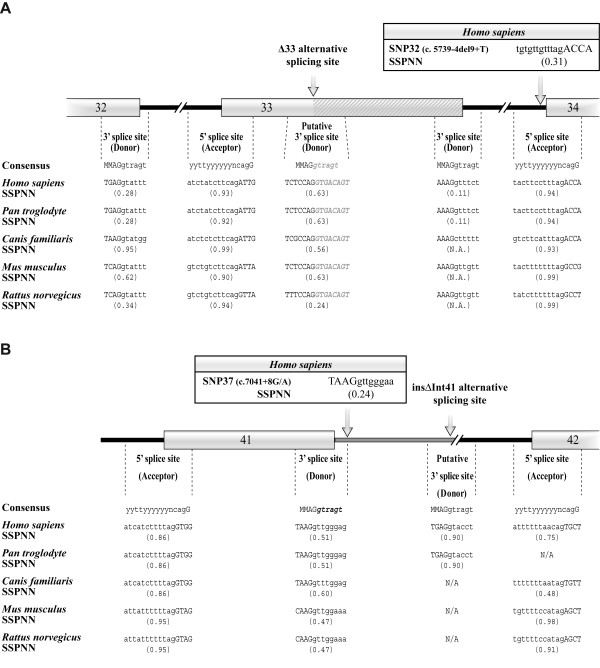
Panel A: Comparative analysis of splicing site sequences in *ATR *exon 32, 33, Δ33 and 34 flanking exon-intron junctions as well as prediction of impact of SNP32 (c.5739-4del9+T) on exon 34 acceptor site. Panel B: Comparative analysis of splicing site sequences in *ATR *exon 41, insΔInt41 and exon 42 flanking exon-intron junctions as well as prediction of impact of SNP37 (c.7041+8G/A) on exon 41 donor site. Splice Site Prediction Program using Neural Network (SSPNN) score values are indicated in parenthesis below each sequence. Exonic nucleotides are represented by uppercase letters while intronic sequences are represented by lowercase letters. m = c or a, r = a or g, y = t or c and n = any nucleotide. N.A. = value not available. Consensus sequences of acceptor and donor sites were described by Burge et al. 1999 [84].

The possible effect on splicing of all coding or intronic variants located in a splice junction was assessed using the SSPNN website and revealed that the intronic variant c.5739-4del9+T (SNP32) located 4 nucleotides upstream of exon 34 decreased exon 34 consensus acceptor site score from 0.94 to 0.31 (Figure 4, Panel A). The presence of the intronic variant c.5739-4del9+T, located in intron 33, became of interest regarding its possible implication to generate a new alternative ATR transcript. Acceptor and Donor splice site sequences in this region are well conserved between mammalian species as illustrated in Figure 4. Moreover it should be noted that the *in silico *analysis revealed a weak donor splice site score (0.11) for exon 33 while a putative donor site with a higher splicing score (0.63) was predicted in exon 33, potentially generating an alternative exon.

Another rare variant (c. 7041+8G>A) showed a significant alteration in the splicing score for a donor site, leading to a score from 0.51 to 0.24 (Figure 4, Panel B). *In silico *analysis of the region surrounding the c.7041+8G/A variant revealed that a putative intronic donor site located 441 nucleotides downstream of the exon 41 could be alternatively used instead of the exon 41 donor site. The WT exon 41 donor site found in other species displays a weak splicing score. The intronic putative donor site located in intron 41 has a higher splicing score in human (0.90) and *Pan troglodyte *(0.90), while no corresponding sequence could be identified in other species analyzed.

### Assessment of the presence of alternative transcripts

**Figure 5:**
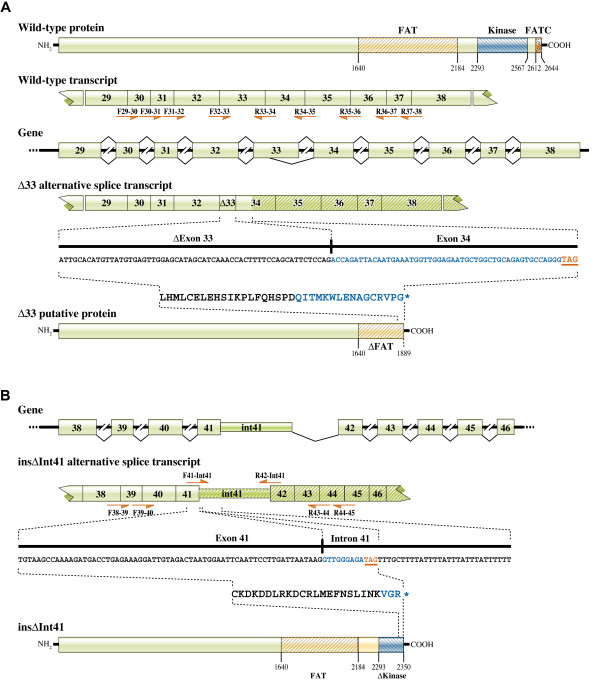
Panel A: Alternative splicing of *ATR *Δ33 exon. Schematic representation of the design used to assess the existence of mRNA splice transcript encompassing exons 33 and 34 in immortalized cell lines of individuals affected with breast cancer. The putative truncated ATR protein of 1889 amino acids lacks the functional domains identified in the wild type ATR protein. Panel B: Alternative splicing of *ATR *insΔInt41 exon. Schematic representation of the design used to assess the existence of mRNA splice transcript encompassing exons 41 and 42 in immortalized cell lines of individuals affected with breast cancer. The putative truncated ATR protein of 2350 amino acids lacks a part of the kinase domains identified in the wild type ATR protein.

**Figure 6:**
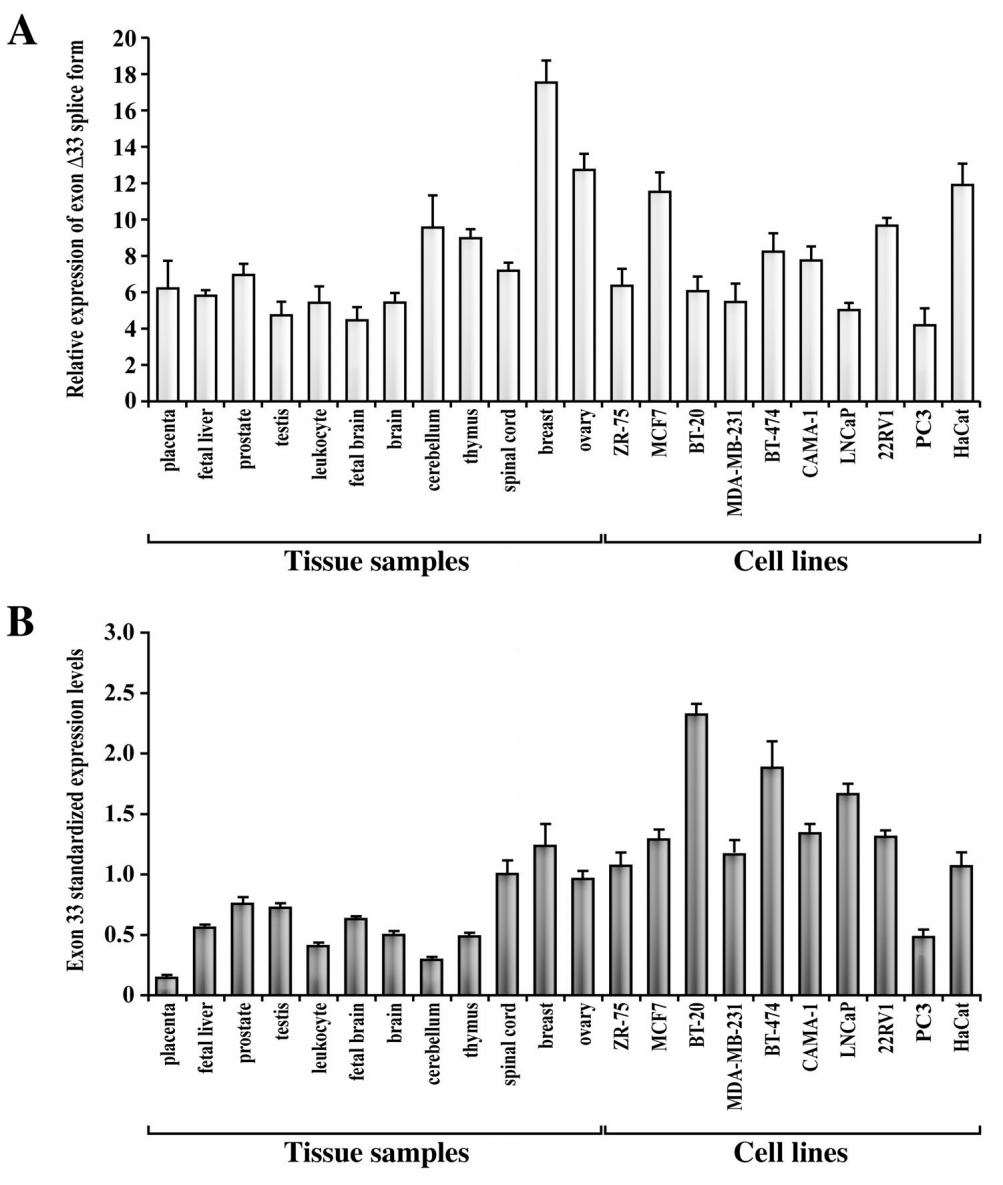
Expression levels of ATR Δ33 splice form in cell lines and human tissues as measured by quantitative real-time PCR experiments. Panel A. Relative expression levels of Δ33 splice form were calculated as Δ33 splice form/(Δ33 splice form + wild-type allele) in various human tissues and cell lines. Panel B. Standardized expression levels of WT exon 33 were calculated as WT exon 33/(Δ33 splice form + wild-type allele) in various human tissues and cell lines.

Following direct sequencing of cDNA PCR products in the region surrounding the c.5739-4del9+T and the c.7041+8G/A variants, we found a novel alternative splice mRNA (Δ33 splice form) showing a deletion of the last 121 nucleotides at the end of exon 33 (Figure 5, Panel A). The deletion causes a shift in the open reading frame (ORF) creating a premature stop codon in exon 34, yielding a putative truncated protein of 1889 amino acids which lacks the C-terminal part of the FAT domain, the entire kinase catalytic domain as well as the FATC domain. Subcloning of PCR products covering this region confirmed the presence of this Δ33 splice form in the immortalized lymphoblastoid cell lines from our breast cancer cases (Figure 6). Analysis through the UCSC genome website revealed the presence of two human ESTs corresponding to the Δ33 splice transcript sequence (BG770191 and CD642306), supporting the presence of such a transcript in humans. Interestingly this alternative transcript uses the putative 3' splicing site predicted in exon 33 described above, which is conserved in most of the mammalian species analyzed. In order to confirm that no additional alternative transcripts involving the skipping of exon 34 could be generated due to this c.5739-4del9+T variant, we used specific primers located on putative exon 33–35 and Δ33-35 junctions in combination with upstream and donwstream primers for PCR amplification. No such detectable PCR product was observed. Besides, no EST corresponding to this potential transcript was found in the UCSC genome website.

No alternative splice transcript has been identified in the region comprising the c.7041+8G/A variant using standard procedures such as PCR amplification with external primers located on exon 38–39, 39–40, 43–44 and 44–45 junctions, followed by subcloning. However, PCR amplification using a specific primer located on a putative exon41-intron41 junction or another primer located 441 nucleotides downstream of exon 41 on the putative Intron41-Exon42 junction identified by *in silico *analysis as described above, revealed the presence of such an alternative transcript (Figure 5, Panel B). However, this alternative transcript is not reported in UCSC database. Since preliminary expression analyses performed in immortalized cell lines of an c.7041+8G/A heterozygote carrier and 9 wild-type individuals revealed an expression at the limit of detection (data not shown), no further expression analyses have been performed.

### Characterization of Δ33 splice mRNA expression

Further characterization of expression levels of Δ33 splice form was performed in several normal tissues and cancer cell lines. As shown in panel A (Figure 6), relative expression levels of Δ33 splice form are highest in the breast and ovary, with relative expression levels of approximately 18% and 13% of the wild-type full-length splice form, while other examined tissues showed similar and lower expression levels. In breast cancer cell lines, Δ33 splice form expression ranges from approximately 6% to 11% in relation to total exon 33 expression (wild-type + Δ33 splice form) and no significant variation is observed according to estrogen receptor or differentiation status. ATR wild-type expression levels standardized for 18S RNA levels are also illustrated in panel B of Figure 6, and show variable expression across tissue samples and somewhat slightly higher expression levels in cancer cell lines.

### Is the c.5739-4del9+T variant associated with Δ33 splice transcripts?

**Figure 7:**
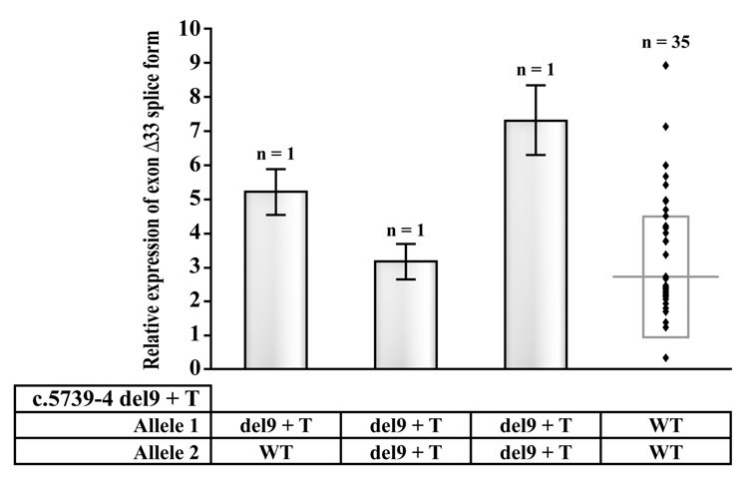
Expression levels of Δ33 splice form in homozygote, heterozygote and wild-type individuals for c.5739-4del9+T variant as measured by quantitative real-time PCR experiments. For the 35 wild type individuals, the confidence interval is represented by an open box with the median indicated.

Assessment of association between the c.5739-4del9+T variant and the expression of the Δ33 splice form was performed using real-time PCR in RNA samples obtained from lymphoblastoid cell lines of 38 of the screened cases, which included 35 wild-type individuals, one heterozygote and two homozygotes for the c.5739-4del9+T variant. As illustrated in Figure 7, the presence of the Δ33 splice transcript was detected in all individuals, including wild-type individuals, therefore supporting that the expression of this splice form is not associated with the presence of the intronic c.5739-4del9+T variant. Furthermore, more interestingly, expression levels do not seem to correlate with genotype status, as observed in heterozygous and homozygous individuals, and high expression variability is also observed in wild-type individuals, whose mean expression level was 2.733 ± 1.806.

Since no evidence suggested that the c.5739-4del9+T variant could be associated with the presence or the expression of the Δ33 splice form, amplification of several cDNA fragments covering exons 30–38 using different combinations of primers located on exon-exon junctions (Figure 5) was performed to detect any additional splice form resulting from the effect of this sequence change on mRNA splicing. No additional splice mRNA was observed.

## Discussion

Since it is well established that the residual familial risk of breast cancer, not caused by *BRCA1 *or *BRCA2 *genes, could be explained by a polygenic or high-risk genes heterogeneity model [72,73], we selected individuals affected with breast cancer without mutations in *BRCA1/2 *genes from high-risk families (one individual per family), in order to increase the power of the study to find genetic variants involved in breast cancer susceptibility. So far, several genes have been investigated based on their interaction with *BRCA1/2 *or their involvement in DNA repair mechanisms. Since *BRCA1/2 *genes are intimately linked to genomic stability, other genes involved in this pathway are very good candidates to be *BRCA3*, and this is especially true of *ATM *and *ATR *which play a central role in genome stability maintenance. The *ATM *gene has been suspected to be a breast cancer susceptibility locus, due to the presence of breast cancer in A-T families, particularly among *ATM *heterozygotes [74]. *ATM *mutations have already been reported to increase breast cancer susceptibility [9,27,75], while some other sequence variants located in this gene do not seem to be linked to breast cancer [24].

Based on the similar roles played by ATM and ATR as sensors of DNA damages, *ATR *may be considered a putative candidate gene that could possibly explain a fraction of the remaining familial breast cancer risk. Association of *ATR *germline mutation with breast cancer susceptibility has been previously analyzed in Finnish 126 families [70], and no germline mutation was identified in this founder population. The current study, performed in a French Canadian cohort, also being a founder population, was designed to assess the possible involvement of *ATR *germline deleterious mutations in breast cancer predisposition.

No deleterious germline mutation leading to a premature termination of the protein were identified in the coding region. However, 41 sequence variants were identified, among which 16 were coding variants while 21 were novel changes. In addition we find it unlikely that neither of the common missense substitutions located in the FAT and kinase domains (c.6394T>G and c.7274G>A) have a significant effect on protein function because: (i) their frequencies are similar in cases and controls, especially for c.7274G>A whose MAF is greater than 20% in controls and (ii) these residues are not well conserved in other species (Table 4). Indeed, the polymorphisms displaying a significant deviation from HWE are composed of a group of 14 uncommon polymorphisms identified in the same 3 breast cancer cases (2 homozygotes and 1 heterozygote), and therefore this most likely constitutes a single relatively rare allele. It has to be stated that no particular characteristics seem to emerge for the families bearing any of these rare variants, as both the French Canadian and the validation families have been recruited on the basis of high-risk breast cancer families.

Comparison of polymorphism frequencies between our cohort and the Finnish cohort [70] is not fully informative since the latter does not distinguish the number of heterozygotes and homozygotes found in their cohort but only the number of carriers of a given polymorphism. However, if we also use this method to calculate polymorphism frequencies observed in our cohort, only SNP40 displayed a notably lower frequency than that found in the Finnish cohort. As stated earlier, both studies (Heikkinen *et al*. and the present study) have been designed to identify *ATR *deleterious germline mutations in breast cancer cases. No such mutation was found in either study, therefore *ATR *is unlikely to play a major role as a high penetrance gene in breast cancer predisposition. Even though novel variants have been identified, the possible involvement of polymorphisms or haplotypes observed in cases compared to those found in controls would need a lot more individuals to obtain a significant value of association to breast cancer susceptibility [76,77]. We thus sought to identify tSNPs that could be useful to other studies and populations.

Our pairwise linkage disequilibrium analysis (Figure 2) did not seem to identify any distinct LD blocks within *ATR*. This observation is supported by the fact that SNP1 is in perfect LD with most other SNPs, including the most distal SNP41, and is also in accordance with what is seen in the French Canadian founder population which displays large conserved haplotypes as reported at the *BRCA1 *locus [49]. However, using the Haploview software, three distinct LD blocks were identified at the *ATR *locus when using SNPs showing a MAF >5% in healthy French Canadian individuals (Figure 3). The breakage of strong LD seems to be located in the region of exon 31, and between exon 43 and exon 47.

Based on the same algorithm (Haploview), and using the SNPs genotyped in HapMap database showing a MAF higher than 5%, two LD blocks could be identified; the first block comprising the SNPs located from intron 1 to exon 43, while the second block included all the remaining SNPs until exon 47. However, it should be noted that the majority of the SNPs used to determine haplotype blocks have a MAF higher than 0.4, which represent common SNPs found in many different populations and therefore probably exclude the SNPs specifically observed in our French Canadian founder population.

We were able to demonstrate that 8 tSNPs are sufficient to represent the majority of *ATR *haplotypes in our French Canadian individuals, which will greatly facilitate subsequent studies. Our results of 8 tSNPs at the *ATR *locus in our population is consistent with previously reported number of tSNPs required at other gene loci in other populations [78,79]. We can therefore be quite confident that these tSNPs will be useful in subsequent analyses. Moreover, out of 72 SNPs genotyped in the HapMap database (HapMap data rel#20 on NCBI B35 assembly, dbSNP b125) at the *ATR *locus, only 40 displayed a MAF >5%. Among them, 7 tSNPs were identified, 3 of which have been identified as tSNPs in our analyses (rs10804682, rs2229032, rs1802904). Of the remaining four tSNPs identified in HapMap database (rs11920625, rs9856772, rs6805118 and rs9816736), three have not been genotyped in our cohort as they were located in intronic regions (>150–200 bp) and one (rs11920625) was not observed in our individuals.

Sequence analysis of exon 34 flanking intronic sequences revealed a deletion of 9 nucleotides + insertion T (c.5739-4del9+T), which is located 4 nucleotides upstream of this exon. *In silico *analysis showed that this deletion decreased the exon 34 acceptor site splicing score from 0.94 to 0.31, which suggests potential splicing alteration in this region.

Surprisingly, sequence analysis of this cDNA region in our immortalized cell lines revealed a deletion of the last 121 nucleotides of exon 33 instead of a skipping of exon 34 (or a portion of exon 34), as expected. This deletion of 121 nucleotides alters the ORF and results in a putative truncated protein of 1889 amino acids. Although interesting, this deletion is observed at similar levels in all tested individuals and is therefore unlikely related to the c.5739-4del9+T polymorphism. This Δ33 splice form may be explained by the weak wild-type donor site score of exon 33 (0.11) and the presence of an additional donor site located within exon 33, which exhibits a score of 0.63. No splice form involving the skipping of exon 34 has been identified when using specific primers located on the putative Δ33-35 or 33–35 exon junctions.

Splicing score analyses of exon 41 flanking intronic sequences were also analyzed since c.7041+8G/A could potentially affect the splicing in this region. While the exon 41 donor splice site showed a relative low splicing score in all species, the putative intronic donor site (splicing score of 0.90) located 441 nucleotides downstream of exon 41 became of interest, given its potential effect on splicing in this region (Figure 4). The insΔInt41 splice form could not be detected using standard procedures. However, this splice form has been amplified and subcloned by using specific primers located on this putative exon junction, demonstrating its very low mRNA expression (at the limit of detection). Due to this low expression, it was impossible to conclude whether or not this insΔInt41 splice form is associated with the c.7041+8G/A variant.

The ratio of Δ33 splice form/WT form being a potentially important factor regarding DNA repair and other related functions in genome stability, we performed QRT-PCR to estimate the relative abundance of WT and Δ33 splice form mRNAs, using TAQMAN probes to allow discrimination between both forms. No correlation was found between the presence of c.5739-4del9+T in either the heterozygous or homozygous state and the expression levels of the Δ33 splice form. However, it is very interesting that significant relative expression of the Δ33 splice mRNA is observed in breast and ovarian tissues, as well as in MCF7 and HaCat (human skin keratinocytes) cells (Figure 6A), especially since expression levels of the WT ATR form in these tissues (Figure 6B) seem to be relatively similar to other tissues and cell lines. The ratio of expression levels between both mRNAs could therefore be of primary issue regarding the effect of the balance of these transcript levels on cell integrity in different human tissues. However it should be noted that only one sample per tissue was analyzed, which by no means represents a mean expression in these tissues or cell lines. While alternative splicing within the non-catalytic domain of ATR mRNA transcript causing skipping of exon 6 had already been observed [80], in 2003 O'Driscoll and coll. [81] identified a founder mutation (2101A→G) in *ATR *that affects exon 9 splicing in two related Pakistani families affected with Seckel syndrome. This study also shows an impaired response to DNA damage in a cell line from an affected parent who carried the mutation. Further characterization of ATR-Seckel cells showed impaired phosphorylation of ATR-dependent substrates, impaired G2/M checkpoint arrest and supernumery centrosomes in mitotic cells, clearly demonstrating a role for ATR in the maintenance of centrosome stability [82]. More recently, two other splicing alterations of ATR have been reported in clinical samples with pyothorax-associated lymphoma [83].

## Conclusion

No deleterious germline mutations have been identified in French Canadian breast cancer cases. However, we have conducted the first detailed haplotype tagging analysis of the *ATR *gene within control individuals from the French Canadian population. The data presented here clearly identified 8 *ATR *tSNPs, which will be useful for other large-scale association studies. We did not find any germline mutations in the *ATR *gene potentially involved in breast cancer predisposition. However, given that different splicing alterations of ATR have been associated with impaired response to DNA damage, the notably significant expression of the novel Δ33 splice form observed in breast and ovarian tissues could have a potential effect on DNA repair mechanisms in these cells, although exhaustive analyses should be required to verify this hypothesis. Further analyses in other populations and larger cohorts will be required to define the possible association of *ATR *gene polymorphisms with breast cancer susceptibility.

## Abbreviations

ATR: Ataxia Telangiectasia-mutated and Rad3-related

CV/CD: Common Variant/Common Disease

ATM: Ataxia Telangiectasia-mutated

PIK: Phosphatidyl Inositol-Kinase

ATRIP: ATR-Interacting Protein

MRN: Mre11/Rad50/NBS1

Smc1: Structural Maintenance of Chromosome 1

MLPA: Multiplex Ligation-dependant Probe Amplification

LD: Linkage Disequilibrium

tSNPs: Tagging SNPs

ATCC: American Type Culture Collection

ER: Estrogen Receptor

QRT-PCR: Quantitative RT-PCR

C_T _: Threshold Cycle number

SSPNN: Splice Site Prediction Program using Neural Networks

dbSNP: Single Nucleotide Polymorphism database

HWE: Hardy-Weinberg Equilibrium

FAT: Frap/ATM/TRRAP

PI3Kc: Phosphoinositide 3-Kinase related catalytic

ORF: Open reading Frame

## Competing interests

The author(s) declare that they have no competing interests.

## Authors' contributions

FD and JS conceived and devised the overall strategy for this study and authored the final version of this manuscript. YL and PS performed all DNA sequence and database analyses and drafted the manuscript. Haplotype analyses were carried out by YL while RNA expression analyses were carried out by PS. OS and ST provided insightful comments and revisions of the final version of the text and carried out DNA sequencing of the patients of the validation group. DL provided DNA samples from healthy patients. PB, JC, RL, JP, BL, RP and MP are clinicians that were in charge of collecting blood samples form affected individuals in their respective institutions and have been highly involved throughout the whole recruitment process to result disclosure. All authors read and approved the final manuscript.

## Pre-publication history

The pre-publication history for this paper can be accessed here:



## Supplementary Material

Additional File 1 Oligonucleotide primers used for amplification and sequence analysis of the ATR gene. The table provides the primers used for amplification and sequencing of the ATR gene. Click here for file

## Figures and Tables

**Table 1:** Observed coding and intronic sequence variants and genotype frequencies in familial breast cancer cases

SNP Designation					Genotype Frequency			
				
SNP	SNP ID^1^	dbSNP ID	Amino acid change	Location	Common homozygote (expected)^2^	Heterozygote (expected)^2^	Rare homozygote (expected)^2^	MAF
1	c.160-179deIT	N/A	-	Intron 1	0.94 (0.91)	0.02 (0.09)	0.04 (0.00)	0.046
2	c.60-51A>T	N/A	-	Intron 1	0.81 (0.82)	0.19 (0.17)	0.00 (0.01)	0.093
3	c. 293-43G>A	N/A	-	Intron 3	0.94 (0.90)	0.02 (0.10)	0.04 (0.00)	0.049
4	c. 293-19C>T	N/A	-	Intron 3	0.94 (0.94)	0.06 (0.06)	0.00 (0.00)	0.029
5*	c.632C>T	rs2227928	Thr211Met	Exon 4	0.42 (0.38)	0.38 (0.47)	0.19 (0.15)	0.385
6	c. 946G>A	rs28897764	Va131611e	Exon 4	0.94 (0.90)	0.02 (0.10)	0.04 (0.00)	0.047
7	c. 1326A>G	rs28897765	Lys442Lys	Exon 5	0.94 (0.91)	0.02 (0.09)	0.04 (0.00)	0.046
8	c. 1488C>T	N/A	Val496Val	Exon 6	0.98 (0.98)	0.02 (0.02)	0.00 (0.00)	0.010
9	c. 1732+I66A>G	N/A	-	Intron 7	0.98 (0.98)	0.02 (0.02)	0.00 (0.00)	0.009
10	c. 1732+279A>G	N/A	-	Intorn 7	0.94 (0.94)	0.06 (0.06)	0.00 (0.00)	0.028
11	c. 1776A>T	rs2227930	Gly592Gly	Exon 8	0.40 (0.34)	0.38 (0.49)	0.23 (0.17)	0.415
12	c. 1815T>C	rs2227929	Asp605Asp	Exon 8	0.40 (0.35)	0.40 (0.48)	0.21 (0.17)	0.406
13	c. 1950G>A	rs28910270	Glu650Glu	Exon 9	0.94 (0.91)	0.02 (0.09)	0.04 (0.00)	0.047
14	c.2290A>G	N/A	Lys764Glu	Exon 10	0.98 (0.98)	0.02 (0.10)	0.00 (0.00)	0.010
15	c. 2634-74T>C	rs9869842	-	Inton 12	0.39 (0.34)	0.39 (0.49)	0.22 (0.17)	0.417
16	c. 2875G>A	rs28910271	Val959Met	Exon 14	0.94 (0.90)	0.02 (0.10)	0.04 (0.00)	0.052
17	c. 2977-97T>C	N/A	-	Intron 14	0.94 (0.91)	0.02 (0.09)	0.04 (0.00)	0.048
18	c. 3120G>A	rs28910272	Leu1040Leu	Exon 15	0.94 (0.91)	0.02 (0.09)	0.04 (0.00)	0.048
19	c.3172-26T>A	N/A	-	Intron 15	0.94 (0.91)	0.02 10.09)	0.04 (0.00)	0.046
20	c.3357+128G>A	rs13091637	-	Intron 16	0.50 (0.51)	0.43 (0.41)	0.07 (0.08)	0.287
21	c. 3725+51T>G	N/A	-	Intron 19	0.98 (0.98)	0.02 (0.02)	0.00 (0.00)	0.009
22	c. 3726-47A>G	N/A	-	Intron 19	0.76 (0.74)	0.20 (0.24)	0.04 (0.02)	0.139
23	c.3945+171C>T	N/A	-	Intron 21	0.96 (0.93)	0.00 (0.07)	0.04 (0.00)	0.038
24	c. 3946-48C>A	N/A	-	Intron 21	0.94 (0.91)	0.02 (0.09)	0.04 (0.00)	0.046
25	c. 4383-232T>G	rs9855919	-	Intron 24	0.43 (0.38)	0.36 (0.47)	0.21 (0.15)	0.387
26	c.4383-177C>T	rs10804682	-	Intron 24	0.72 (0.74)	0.28 (0.24)	0.00 (0.02)	0.139
27	c. 5208T>C	rs2227931	Tyr1736Tyr	Exon 30	0.38 (0.32)	0.37 (0.49)	0.25 (0.19)	0.441
28	c. 5288+130G>A	rs11916455	-	Intron 30	0.44 (0.26)	0.31 (0.48)	0.25 (0.16)	0.403
29	c. 5380+121C>A	rs11927681	-	Intron 31	0.87 (0.87)	0.13 (0.13)	0.00 (0.00)	0.065
30	c. 5460T>C	rs2227932	Tyr1820Tyr	Exon 32	0.79 (0.78)	0.19 (0.21)	0.02 (0.01)	0.115
31	c. 5558+119C>T	N/A	-	Intron 32	0.96 (0.92)	0.00 (0.08)	0.04 (0.00)	0.038
32	c. 5739-4dc19+T	N/A	-	Intron 33	0.94 (0.91)	0.02 (0.09)	0.04 (0.00)	0.046
33	c.5868C>T	N/A	Tyr1956Tyr	Exon 34	0.96 (0.93)	0.00 (0.07)	0.04 (0.00)	0.038
34	c. 6394T>G	rs28910273	Tyr2132Asp	Exon 38	0.98 (0.98)	0.02 (0.02)	0.00 (0.00)	0.009
35	c. 6553-45C>G	N/A	-	Intron 38	0.98 (0.98)	0.02 (0.02)	0.00 (0.00)	0.010
36	c. 6897+88A>G	N/A	-	Intron 40	0 98 (0 98)	0.02 (0.02)	0.00 (0.00)	0.009
37	c. 7041+8G>A	N/A	-	Intron 41	0.98 (0.98)	0.02 (0.02)	0.00 (0.00)	0.009
38	c. 7041+55T>A	N/A	-	Intron 41	0.98 (0.98)	0.02 (0.02)	0.00 (0.00)	0.009
39	c. 7274G>A	rs2229032	Arg2425Gln	Exon 43	0.75 (0.73)	0.02 (0.25)	0.04 (0.02)	0.147
40	c. 7875A>G	rs1802904	Gln2625Gln	Exon 47	0.80 (0.77)	0.16 (0.22)	0.04 (0.01)	0.120
41	c. 7932+104T>C	rs10935463	-	Intron 47	0.39 (0.33)	0.37 (0.49)	0.24 (0.18)	0.422

^1^According to the nomenclature of the Human Genome Variation Society

^2^As expected under Hardy-Weinberg equilibrium

N/A: Information not available for this SNP (not reported in dbSNP)

T allele is considered as the common allele according to the reference sequence NM_001184

MAF : Minor Allele Frequency

**Table 2:** ATR sequence variant allele frequencies in different cohorts

			French-Canadian families		Validation group		Finnish families^2^		Reported in NCBI database
SNP	SNP ID^1^	dbSNP ID	*n*	MAF	*n*	MAF	*n*	Carrier frequency	MAF

-	C.268C>T	N/A	51	0.000	-	-	126	0.127	N/A
5*	C.632C>T	rs2227928	52	0.385	45	0.389	126	0.460	0.425
6	c.946G>A	rs28897764	50	0.047	42	0.037	126	0.000	N/A
11	C.1776A>T	rs2227930	53	0.415	-	-	126	0.508	0.412
12	C.1815T>C	rs2227929	53	0.406	-	-	126	0.468	0.378
14	c.2290A>G	N/A	50	0.010	41	0.000	126	0.000	N/A
16	c.2875G>A	rs28910271	48	0.052	46	0.000	126	0.000	0.010
27	c.5208T>C	rs2227931	49	0.441	-	-	126	0.238	0.263
30	c.5460T>C	rs2227932	52	0.115	-	-	126	0.262	0.105
32	c. 5739-4del9+T	N/A	54	0.046	42	0.012	126	0.000	N/A
34	C.6394T>G	rs28910273	54	0.009	44	0.000	126	0.008	0.016
39	c.7274G>A	rs2229032	51	0.147	41	0.170	126	0.278	0.109
40	c.7875A>G	rs1802904	50	0.120	-	-	126	0.270	0.101

^1 ^According to the nomenclature of the Human Genome Variation Society

^2 ^Heikkinen *et al*. 2005, Breast Cancer Research 7 :R495-R501

N/A: Information not available for this SNP or not reported in dbSNP

- Not genotyped

* T allele is considered as the common allele according to the reference sequence NM_001184

MAF : Minor Allele Frequency

**Table 3:** Coding and intronic (MAF>5%) sequence variations and genotype frequencies in familial breast cancer cases and controls

SNP	SNP ID^1^	Series	# individual	Common homozygote No. (expected)^2^	Heterozygote No. (expected)^2^	Rare homozygote No. (expected)^2^	MAF^3^	X^2 ^p-value^4^
2	c.60-51A>T	Cases	54	44 (44.3)	10 (9.2)	0 (0.5)	0.093	0.453
		Controls	62	49 (47.9)	11 (13.2)	2 (0.9)	0.121	0.192
5*	c.632C>T	Cases	52	22 (19.8)	20 (24,4)	10 (7.8)	0.385	0.176
		Controls	71	26 (29.8)	40 (32.4)	5 (8.8)	0.344	0.048
6	c.946G>A	Cases	53	50 (48.1)	1 (4.7)	2 (0.1)	0.047	<0.001
		Controls	68	68 (68.0)	0 (0.0)	0 (0.0)	0.000	-
7	c.1326A>G	Cases	54	51 (49.1)	1 (4.9)	2(0.0)	0.046	<0.001
		Controls	72	68 (68.1)	4 (3.9)	0(0.1)	0.028	0.808
8	c.1488C>T	Cases	51	50 (50.0)	1 (1.0)	0 (0.0)	0.010	0.944
		Controls	73	73 (73.0)	0 (0.0)	0(0.0)	0.000	-
11	c.1776A>T	Cases	53	21 (18.0)	20 (26.0)	12 (9.0)	0.415	0.105
		Controls	70	25 (29.6)	41 (31.8)	4 (8.6)	0.350	0.016
12	c.1815T>C	Cases	53	21 (18.5)	21 (25.5)	11 (9.0)	0.406	0.194
		Controls	71	25 (25.5)	35 (34.1)	11 (11.4)	0.401	0.828
13	c.1950G>A	Cases	53	50 (48.2)	1 (4.8)	2(0.0)	0.047	<0.001
		Controls	71	68 (68.0)	3 (2.9)	0(0.1)	0.021	0.856
14	c.2290A>G	Cases	50	49 (49.0)	1 (1.0)	*a *(0.0)	0.010	0.943
		Controls	72	72 (72.0)	0 (0.0)	0 (0.0)	0.000	-
15	c.2634-74T>C	Cases	54	21 (18.3)	21 (26.3)	12 (9.4)	0.417	0.142
		Controls	51	15 (18.2)	31 (24.6)	5 (8.2)	0.402	0.059
16	c.2875G>A	Cases	48	4S (43.2)	1 (4.8)	2(0.0)	0.052	<0.001
		Controls	68	64(64.0)	4 (3.9)	0(0.1)	0.028	0.803
18	c.3120G>A	Cases	52	49 (47.3)	1 (4.7)	2 (0.0)	0.048	<0.001
		Controls	70	66(66.0)	4 (3.9)	0(0.1)	0.028	0.806
20	c.3357+128G>A	Cases	54	27 (27.5)	23(22.1)	4(4.4)	0.287	0.765
		Controls	64	41 (40.7)	20 (20.7)	3 (2.6)	0.203	0.781
22	c.3726-47A>C	Cases	54	41 (40.0)	11 (13.0)	2 (1.0)	0.139	0.275
		Controls	65	44(43.2)	18(19.6)	3(2.2)	0.185	0.518
25	c.4383-232T>G	Cases	63	33 (19.9)	19 (25.2)	11 (7.9)	0.387	0.013
		Controls	65	23 (25.9)	36 (30.3)	6(8.8)	0.369	0.127
26	c.4383-177C>T	Cases	54	39(40.0)	15(13.0)	0(1.0)	0.139	0.236
		Controls	65	37(37.0)	24 (24.1)	4(3.9)	0.246	0.967
27	c.5208T>C	Cases	51	39 (16.3)	19 (25.0)	13 (9.7)	0.441	0.081
		Controls	65	23 (23.4)	32 (31.2)	10(10.4)	0.400	0.836
28	c.5288+130G>A	Cases	36	16 (12.9)	11 (17.3)	9(5.8)	0.403	0.028
		Controls	55	20 (21.7)	29 (25.7)	6(7.6)	0.372	0.344
29	c.5380+121C>A	Cases	46	40 (40.2)	6 (5.6)	0(0.2)	0.065	0.636
		Controls	62	JO (48.8)	10 (12.4)	2 (0.8)	0.113	0.125
30	c.5460T>C	Cases	52	41 (40.1)	10(11.4)	1 (0.5)	0.115	0.676
		Controls	72	62 (62.4)	10 (9.3)	0(0.3)	0.069	0.526
32	c.5739-4del9+T	Cases	54	51 (49.1)	1 (4.9)	2(0.0)	0.04	<0.001
		Controls	63	60 (60.0)	3 (2.9)	0 (0.0)	0.024	0.846
33	c.5868C>T	Cases	53	51 (49.3)	0 (3.7)	2 (0.0)	0.038	<0.001
		Controls	67	67(67.0)	0 (0.0)	0 (0.0)	0.000	-
34	c.6394T>G	Cases	54	53 (52.9)	1 (1.1)	0(0.0)	0.009	0.945
		Controls	66	66 (66.0)	0 (0.0)	0 (0.0)	0.000	-
37	c.7041+8G>A	Cases	53	52(52.1)	1 (0.9)	0 (0.0)	0.009	0.945
		Controls	70	65 (63.2)	3 (6.6)	2(0.2)	0.050	<0.001
39	c.7274G>A	Cases	51	38(37.2)	11 (12.8)	2 (1.0)	0.147	0.317
		Controls	70	39(40.9)	29 (25.2)	2(3.9)	0.236	0.210
40	c.7875A>G	Cases	50	40(38.5)	8 (11.0)	2 (0.5)	0.120	0.086
		Controls	63	45 (45.4)	17 (16.1)	1 (1.5)	0.151	0.670
41	c.7932+104T>C	Cases	51	20 (17.0)	19 (24.9)	12 (9.1)	0.422	0.009
		Controls	53	19 (18.7)	25 (25.6)	9(8.7)	0.406	0.874

^1 ^According to the nomenclature of the Human Genome Variation Society

^2 ^As expected under Hardy-Weinberg equilibrium

^3 ^Minor allele frequency

^4 ^p-value for deviation from Hardy-Weinberg equilibrium (Pearson's chi-square)

* T allele is considered as the common allele according to the reference sequence NM_001184

**Table 4:** Sequence variants detected in Human ATR and residues found in orthologues

SNP	SNP ID*	Amino acid change	*Pan troglodytes*	*Canis familiaris*	*Mus musculus*	*Xenapus laevis*	*Fugu rubribes*	*Strongylocentrotus purpuratus*	*Drosophila melanogaste*
5**	c.632C>T	Thr211 Met	Thr	Thr	Met	Met	Gly	*-*	Glu
6	c. 946G>A	Va1316Ile	Val	Val	Val	Val	Val	Ser	Asp
14	c. 2290A>G	Lys764Glu	Lys	Lys	Lys	Lys	Lys	Ser	Glu
16	c. 2875G>A	Val959Met	Met	Met	Ile	Met	Ser	Thr	Phe
34	c. 6394T>G	Tyr2132Asp	Tyr	His	Arg	Gln	Asn	Lys	Ala
39	c. 7274G>A	Arg2425Gln	-	Arg	Gln	Lys	Lys	Glu	Thr

* According to the nomenclature of the Human Genome Variation Society

- No corresponding residue in this species

** T allele is considered as the common allele according to the reference sequence NM_001184
