# Supplementary material for: Mutation analysis and characterization of ATR sequence variants in breast cancer cases from high-risk French Canadian breast/ovarian cancer families
Source: BMC Cancer. 2006 Sep 29;6:230. doi: 10.1186/1471-2407-6-230 (PMC1599749; doi:10.1186/1471-2407-6-230)
Supplement: Additional File 1 — Oligonucleotide primers used for amplification and sequence analysis of the ATR gene. The table provides the primers used for amplification and sequencing of the ATR gene. [file 1471-2407-6-230-S1.pdf]

Additional file 1

Oligonucleotide primers used for amplification and sequence analysis of the ATR gene

Exon	Forward primer (5'-3')	Reverse primer (5'-3')	Product length (bp)	Annealing Temp (C)
1	ACGAACGTCGCTCGCTCGGT*	ATCAACGGGCACTCTGAGGGAGA	519	58
2	GCTGATTGGATCTCTTTAGTATTACTTAG	AATACTGATTAACAAATAACTTGCTTGA*	458	52
3	CCACTCCCAGCTAATTTTAAGA*	TTGATTATTTCTAAGTACCTAGTATACAATACT	580	52
4	GTATGCCTGTGTTCTAAACTATTATGT*	AAAATCATTATGTTCCAAATATAAATTACT*	1174	50
5	ACTAATTTAGATGTTTATTTTGCTTTATGA*	ATGTTCTTTGTGTATTTAAATCAAAGCA	433	52
6	ATTCTAAATATGTTTCATGTTTTAACCA	ACCGTACCTAGCATTTGACTCA*	427	52
7-8	TCCTAAGGGTGATTTAAGCTGACA*	CAAACCACACACACATTCTTGTGA	1050	52
	ACATATTACTTGATTTGCATGTTTACT*			
	GTTCACAGACCTCAGACCATCGT*			
9-10	GGTATTAAATGTATTTTAAGTGTTACTTGA*	CCAGTTATCCCATTTATAACTAATCA*	999	52
	TCTGGCTATGTATGCAGGGTTGT*			
11-12-13	ATACATACTTAAATATGAAACATTGACA*	TTAAAAAGTACTGCTGTTGCCTA	978	52
	GTGTGTGTGTGTGTGTTTGTGTATTCA*	ACACAAATGCTGCCAAGTATACCT*		
		AAAGTACTGCTGTTGCCTATAGTCCA*		
14-15	GCAGATTATGACATAGTCTACCTCA*	GATAGGCTATAATTTACTCACCCTCT*	1094	58
16	CCATGGGAGACCTACCAGGA	CTATAAGAAAATAAACGTGATCTCGTGT	586	52
	TCCTGTCATCTGGGCTAGAGCT*			
17	TGGAGAAACTTAATTAACCAAGTGT	CTAAAGCTACATCATCCTTACGCT*	510	52
18	GACAAAACAAACTGGCCAAC*	AGTTGGGAAATAGGTATGTAGGTTC	525	52
19	TGTTTGTATATATCATGTGTCTTCAACTA*	CATTTCAATATGTAATTACATGCCAA	399	52
20	GGTTTATTGTAGCACTATTATAGAGTGA	ATGGATTCATGGTAGCATTATCT*	480	52
21	CCATAGACCTGGTTCAAAGCTA*	GAAGACTTACATAAACTGCTGCTGTA	762	52
22	TCATTGCTTCTTTGCCGTACA	AATATAACACCCTAGATGGGGTTCA*	805	52
23	GATTATATTGTAGTCTTGATACCAAACT	GGCAAACTTCGTTGTCTGA	526	56
		AGTCACAAAGCTGAGGCAGT*		
24	TACTGCATGCATAGTGGCCA	TTGGGCCTACTAAAGGCTTTCT	1002	52
		CTCGAACTCCTGACCTCGTGA*		
25	GCATAGTTACCCATAATGAGGAGT*	ACACACATTAAGACCATAGTAATTTTCT	678	52
26	TGTTAACTATTTTCAGCTCTGGAATA*	GTCTACATTTCCTACTAATAGGTAGCCT	529	52
27	ACCTTTTATGTGATGCCTCATCT	CAGTGAAAAGGCCACACTATTAC	1385	54
	GCTGGTCTTGACCTCAGGCA*			
28	GGAGCAAGACTCTCTGAACAATTGT*	CCACATGGATGAGTTAAGTGTCCT	557	52
29	CAGTTTTAGGATCCTGAGGTTCTT	GAGAGGGTTAAATATTTTCCAGACA*	753	52
30	ACTTTAAATGTGTTAGGACATTGATG*	GAGCCTCACCAATAGCCAGT	697	55
31	TAAATGGTGTATCAGAAGTAATTAGTGA*	AATATTTATGTCATATTAAAACCGCT	676	52
32	CTATAATCATGCCATAGCCCTCT	GACATTACAAGAGGACAAACATAAGAT*	607	52
33	AATTTGGGATTCACTGCAAATCC	CGTACAGTTCAGCGAGTCGTGA	1272	56
	CATGCCTGGCTAAAGCACCA*			
34	AGATTCTCTAAACTGGGTAGCTCGACT	GTTCTCTCAGTTGGCCTTTTAAGGA	881	56
	GGTTCTGTATTGGGAACAGAGGCT			
35	ATGAATGAGATGAAGCAATGT*	GTATATTTATTTTGTGATAATCCATATACT	511	52
36	GGAGTTTTCTCCTAAGTTGCGAA	CCTAGAATATGCTAAGACATGTGATAACA*	498	54
37-38	CAGGGAATGTAGAACTAAGTGAGCT	CTTCATTTCATATGGTAAATATATCCAA*	1231	52

		TCCCATGCCCAAGGTTAGGT*		
39	GGCAGATCCCTATAACTTTATAATTC*	TTTAGAAATATTAGAGGGTATTTGGAAG	563	52
40	TAAAATGGGATGATTAGGAAATACTTAG*	CTTAAATTAAAAACAAAGCATAGACTGG	970	52
41	TTGGCCCTAATTCATATATAACTCATC	ATATTTCATTCCTGTTTTTAAGAGTG*	351	52
42	TAATGTTTCAGTAATACCTATAAAGCAATAG	CCTCAAATCCTGTTGGGATG*	730	52
43-44	TGTGATTAATGGATATCACTCTTCC	CAAATTCAGTACTAGCTTTACAGTATCAC*	675	52
45	TGTAATACTGCTTATTCACTGTACTAAACT	GTGACTGCTAATGCCTACGG*	710	52
46	AGTATACACTTAGAGATGGCACATAGAA*	CATAACTGTGAATTGTCTATGGTTAAAC	635	52
47	GTTGAATCAATCTTGCGAGCCTCC	GGCACAACGCCTTGCCCA	764	58
	TCTTACAAAAGGGGTATTGGTCAGT*			

* Used as sequencing primers
